# Randomized evaluation of redo ablation procedures of atrial fibrillation with focal impulse and rotor modulation-guided procedures: the REDO-FIRM study

**DOI:** 10.1093/europace/euac122

**Published:** 2022-09-03

**Authors:** Stefan G Spitzer, John M Miller, Philipp Sommer, Tamas Szili-Torok, Vivek Y Reddy, Georg Nölker, Chris Williams, Anne Sarver, David J Wilber

**Affiliations:** Praxisklinik Herz und Gefäße, 01099 Dresden, and Brandenburg University of Technology Cottbus-Senftenberg, Institute of Medical Technology, 03046 Cottbus, Germany; Krannert Institute of Cardiology, Department of Medicine, Indiana University School of Medicine, Indianapolis, IN 46202, USA; Herz-und Diabeteszentrum NRW, Universitätsklinik der Ruhr-Universität Bochum, Bad Oeynhausen 32545, Germany; Erasmus MC, Rotterdam, 3015 GD, The Netherlands; Helmsley Electrophysiology Center - Icahn School of Medicine at Mount Sinai, New York, NY 10029, USA; Christliches Klinikum Unna, Unna 59423, Germany; Abbott Laboratories, Chicago, IL 60064, USA; Abbott Laboratories, Chicago, IL 60064, USA; Loyola University Chicago, Chicago, 60660, USA

**Keywords:** Recurrent AF, Rotor, Focal Impulse and Rotor Modulation

## Abstract

**Aims:**

*REDO-FIRM* evaluated safety and effectiveness of conventional vs. focal impulse and rotor modulation (FIRM)-guided ablation of recurrent persistent or paroxysmal atrial fibrillation (AF) after an initial AF ablation procedure.

**Methods and results:**

This prospective, multicentre, randomized study included patients with a single prior AF ablation, but with recurrent AF and reconnected pulmonary veins (PVs). Conventional ablation generally included PV re-isolation; however, additional ablation was permitted per physician discretion. In the FIRM arm, beyond PV re-isolation, basket catheter-based FIRM mapping created dynamic animations of putative rotors, which were targeted for ablation. Between May 2016 and July 2019, 269 subjects were randomized, with 243 subjects completing 12-month follow-up. Ablation beyond re-pulmonary vein isolation, the FIRM vs. Conventional arms did not differ significantly: cavo-tricuspid isthmus –9.0% vs. 15.3%, caval vein isolation –1.5% vs. 0.8%, non-PV trigger –2.2% vs. 3.8%, other –11.9% vs. 13.0%. Single procedure 12-month freedom from AF/atrial tachycardia/atrial flutter-recurrence was 63.3% (76/120) vs. 59.0% (72/122) in the FIRM and Conventional arms (*P* = 0.3503). Efficacy was similar in the paroxysmal and persistent AF subgroups (*P* = 0.22 and *P* = 0.48). The 10-day and 12-month safety endpoints were achieved in 93.3% vs. 93.8% (*P* = 0.89) and 88.4% vs. 93.4% (*P* = 0.22) in the FIRM and Conventional arms, respectively.

**Conclusions:**

In *REDO-FIRM*, as compared to standard ablation, FIRM-guided ablation did not provide additional efficacy in redo ablation procedures, but FIRM-guided ablation was equally safe. Additional studies are necessary to identify any potential population able to benefit from FIRM-guided ablation.

**What’s new?**

- Redo-FIRM was the first randomized trial assessing the safety and efficacy of focal impulse and rotor modulation (FIRM)-guided ablation in redo AF patients.
- Single procedure 12-month freedom from atrial fibrillation (AF)/atrial tachycardia/AFL-recurrence was 63.3% (76/120) vs. 59% (72/122) in the FIRM and Conventional arms, respectively (*P* = 0.3503). Efficacy was similar in the paroxysmal and persistent AF subgroups (*P* = 0.22 and *P* = 0.48, respectively); in summary: Redo-FIRM did not provide evidence of additional efficacy of FIRM-guided ablation in redo ablation procedures—compared to standard ablation, but FIRM-guided ablation was equally safe.
- According to initial assumptions (success of 40% vs. 60% in conventional vs. FIRM-guided subjects), the conventional group did much better (59%) than expected.
- The optimal ablation strategy besides Re-pulmonary vein isolation in case of reconnected veins in Redo-patients remains unclear. Further research for this patient group is needed and should especially focus on patients, in whom the PV`s are completely isolated at the time of re-ablation.

## Introduction

Atrial fibrillation (AF) is the most common sustained arrhythmia encountered in clinical practice. Pulmonary vein isolation (PVI) is the cornerstone of ablative treatment for paroxysmal and persistent AF. The optimal ablation strategy beyond Re-PVI for Redo-cases (i.e. patients who have recurrent AF after prior ablation) in paroxysmal and persistent AF remains unclear. A variety of adjunctive ablation strategies besides PVI have been explored in patients with persistent AF: linear ablation, posterior wall ablation, ablation of complex fractionated electrograms, ganglionic plexus ablation, isolation of the left atrial appendage, ablation of low-voltage areas^1^ and stepwise methods incorporating multiple strategies. While incremental benefit has been reported with some of these approaches, other studies have failed to show a benefit—data for implementing these strategies in redo-procedures are lacking. More recent efforts have attempted to identify drivers that perpetuate AF. Previous work has implicated both focal impulses and rotational activity (rotors) as important drivers for AF.^2^ Sites of high-frequency activity are more commonly seen outside the pulmonary vein (PV) region in persistent AF, which may help to explain the lower success rates of PVI in this group.^3^ Non-PV triggers contribute to post-ablation recurrence in both paroxysmal and persistent AF patients. Procedural success rates can be improved if non-PV foci are detected and eliminated.^4^ A novel mapping technology (RhythmView™ workstation in conjunction with the FIRMap™ diagnostic catheter, Abbott Laboratories, IL, USA) based upon the work by Narayan et al^5^ has been developed for analyzing atrial recordings during human AF, finding that >95% of cases demonstrate either a rapidly spinning rotor (small circuit) or very rapid focal impulse formation. In the Conventional vs Focal Impulse and Rotor Modulation (CONFIRM) trial, authors found higher acute and long-term efficacy when focal impulse and rotor modulation (FIRM) guided procedural approach was added to PVI (82.4 vs. 44% freedom from AF at 24 months post procedure). However, less optimistic long-term clinical outcomes have been published as well.^6^ The purpose of this study was to evaluate the safety and effectiveness of conventional vs. FIRM-guided ablation of recurrent persistent or paroxysmal AF after an initial AF ablation procedure.

## Methods

### Recruitment and study design

REDO-FIRM was a prospective, multicentre, randomized study designed to assess the safety and effectiveness of a FIRM-guided repeat RF ablation procedure including PVI vs. a standard PVI procedure for the treatment of persistent and paroxysmal AF after one failed previous PVI. Ablation of any atrial tachycardias (ATs) and/or the cavotricuspid isthmus (CTI) could also be performed in subjects with documented AT and/or Atrial Flutter (AFL). Sample size power calculations are provided in the Supplementary material online, appendix.

Subjects must have had a single prior AF ablation that occurred after January 1, 2013, but not within 3 months prior to study enrolment. Subjects must have had at least one documented episode of spontaneous persistent or paroxysmal AF since their first ablation. A full listing of the inclusion and exclusion criteria is provided in the Supplementary material online, appendix.

Two hundred and sixty-nine (269) subjects were enrolled and randomized (136 FIRM-guided, 133 Conventional), 4 failed an inclusion/exclusion (I/E) criterion and the remaining 265 subjects comprised the intent to treat (ITT) population. The first subject was enrolled on May 20, 2016, and the last subject was enrolled on July 15, 2019. A total of 243 subjects completed the 12-month follow-up period, 121 in the FIRM-guided arm and 122 in the Conventional arm.

### Study objectives and endpoints

#### Primary effectiveness endpoint—12-month success

The primary effectiveness endpoint was defined as freedom from AF/AT/AFL recurrence at 3–12 months post procedure. Freedom from AF/AT/AFL recurrence was defined as no documented episodes >30 s with conventional noninvasive 7-day monitoring. In the case of a cardiac implanted electronic device, freedom from AF/AT/AFL recurrence was defined as no documented episodes >30 s in a 1-week window at the follow-up visits, in addition to any symptomatic episodes with documented episode >30 s. After the 3-month blanking period, if re-ablation for AF was necessary after documentation of recurrent arrhythmia, the patient was considered a primary effectiveness study failure.

#### Primary safety endpoints

Safety was evaluated at both 10 days and 12 months.

Success was defined as freedom from serious adverse events (SAEs) related to the procedure (including any repeat procedures) within 10 days and within 12 months of the initial procedure. The full definition of what was considered an adverse event is included in the Supplementary material online, appendix.

#### Secondary endpoints

Acute effectiveness was evaluated as a secondary endpoint.

The acute success of the FIRM-guided procedure was defined as no evidence of source on RhythmView immediately post procedure with complete signal elimination in the target area. Good contact was ensured by contact force.

### Data management and follow-up

Subject randomization occurred during the procedure, after the following criteria were met:

1. AF was sustained for at least 5 min uninterrupted, AND
2. Confirmation of PV reconnection.

If the subject was in sinus rhythm and one or more of his/her PVs were not isolated, it was acceptable to isolate the vein(s) before induction of AF. If AF could not be sustained for a minimum of 5 min after re-isolation of PVs, the subject was disqualified prior to randomization and enrolment. Treatment group allocations were randomized using a 1:1 ratio to a series of sequential numbers in blocks of 10.

This clinical study was conducted in accordance with Good Clinical Practice, ISO 14155 ethical principles based on the Declaration of Helsinki, the applicable national regulations and Institutional research policies and procedures.

### Device description

The FIRMap™ Basket Catheter (Abbott Laboratories, IL, USA) is a multi-electrode catheter that can capture unipolar electrogram data from either the right or left atria. Using the FIRMap™ Catheter, the RhythmView™ System (Abbott Laboratories, IL, USA) processes 64 electrograms from areas throughout the cardiac chamber to identify the anatomic locations of these electrical patterns. The RhythmView™ Workstation (Abbott Laboratories, IL, USA) provides software tools for graphical display of patient-specific AF sources. Supplementary material online, appendix figure S1 shows the interface of the RhythmView™ 6.1.

### AF ablation procedure

Randomization occurred during the procedure only after confirmation of PV reconnection (at least one vein) using a circular catheter and AF sustained for at least 5 min. Induction of sustained AF could occur by burst pacing at 200 ms for 5 s from the RA and twice from the CS. Patients were randomized to undergo either conventional ablation (Conventional arm), or FIRM-guided ablation followed by conventional ablation (FIRM-guided arm), following standard of care practices for the treatment of AF. Conventional ablation typically consists of PVI, but the following additional ablations were allowed when part of the Investigator’s standard of care practices: RA-CTI line, Non-PV-trigger (if provoked), superior caval vein isolation. Left atrial appendage isolation was not permitted in either arm of the study. Operators were instructed to follow all device IFUs and follow their standard procedures for RF power and duration.

If standard of care included ablation of extra-pulmonary foci, induction of non-PV triggers could occur in two ways: First, isoproterenol infusion (starting at 2 mcg and incrementing up to 20 mcg per standard laboratory protocol) or another sympathomimetic agent (e.g. IV norepinephrine or epinephrine) could have been substituted if it was standard laboratory protocol. Second, cardioversion of spontaneous AF or AF induced by left or right atrial rapid pacing. AF and the subsequent cardioversion could be induced/performed during infusion of isoproterenol so as to maximize the possibility of inducing AF triggers.

Three dimensional electroanatomic maps were created using CARTO (BioSense Webster, Irvine, CA, USA) or EnSite™ NavX™ (Abbott Laboratories, IL, USA) systems. Voltage mapping was strongly recommended. Source arrhythmia diagnosis was performed by obtaining at least 1 minute of continuous electrograms from the 64-pole FIRMap catheter either simultaneously or sequentially in the right and left atria, then transferring these data to the RhythmView system.

### Follow-up

Enrolled subjects returned for follow-up visits at day 10 (phone or in office), after 6 weeks, 3, 6 and 12 months. Following ablation treatment, subjects underwent a 3-month ‘blanking period’ where medical therapy could be optimized and where any of the procedurally-derived arrhythmias that commonly follow ablation procedures were expected to resolve. A diagnostic 12-lead ECG was performed at baseline, discharge, 6 weeks, 3 months, 6 months, 12 months, and unscheduled visits. All subjects also wore a 7-day ambulatory continuous ECG monitor at 3 months, 6 months, 12 months, and unscheduled visits. Screening for adverse events or symptoms of potential procedural complications (e.g. PV stenosis, cardiac tamponade) occurred at every follow-up visit. Quality of life (QoL) surveys (EQ-5D, AFEQT) were administered at baseline, 6 weeks, 3 months, 6 months, and 12 months.

### Statistical analysis

The primary effectiveness endpoint was Single Repeat Procedure Freedom from AF/AT/AFL recurrence in the period from 3 to 12 months after the initial AF ablation procedure. The proportion of successes in each treatment arm was evaluated using both Chi-Square (Binomial, 1 degree of freedom) and by Kaplan-Meier survival estimation. Each evaluation included 95% confidence intervals.

Detailed description of the further statistical analysis is summarized in the Supplementary material online, appendix.

## Results

### Patient characteristics

**Figure 1:**
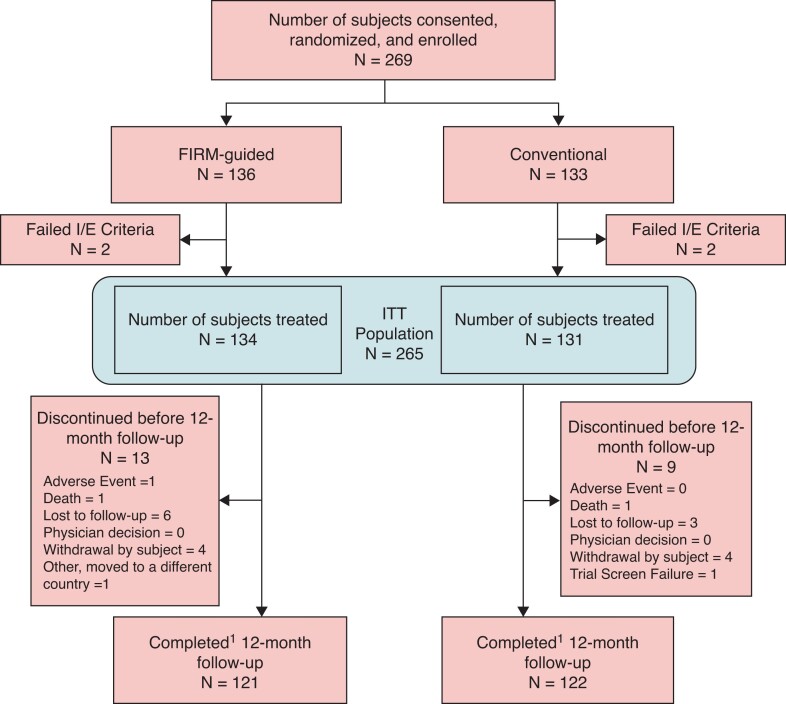
Study flow. ^1^There were 121 focal impulse and rotor modulation-guided subjects and 122 conventional subjects for which the site responded ‘Completed’ to the question ‘Select the reason for the end of the patient’s participation in the STUDY:’ on the Study Exit form. However, for some of these subjects, the site responded ‘Yes’ to the question ‘Did the patient discontinue the study prior to the end of the observation period?’ on the Study Exit form. The site-reported subject disposition was used to determine whether a subject completed the study. Therefore, some subjects are reported as having completed the study without a full 365 days of post-procedure follow-up.

Two hundred and sixty-nine (269) subjects with symptomatic AF were consented and enrolled (‘Enrolled Population’) between May 2016 and July 2019 at 24 different sites in Europe and the United States. Four enrolled subjects failed an I/E criterion and were excluded from the ITT population. Of the 265 ITT subjects, 243 completed the 12-month follow-up period, 121 in the FIRM-guided arm and 122 in the Conventional arm; study flow is shown in *Figure 1*. Subjects that discontinued the study prior to the end of an endpoint observation period without experiencing an endpoint event were excluded from the analysis population of that endpoint.

There were three deaths, two in the FIRM-guided arm (1 subject failed the I/E criterion and was not part of the ITT population) and one in the Conventional arm; none were related to the study procedure or device.

#### Demographics and baseline assessments for study population

Patient demographics for the Enrolled Population are shown in *Table 1*.

**Table 1:** Patient demographics (enrolled population)

	FIRM-guided (*N* = 136)	Conventional (*N* = 133)	*P*-value
**Age (years),** mean ± SD (*n*)	62.8 ± 12.2 (136)	63.2 ± 10.9 (133)	0.7596^a^
**Sex (male)**	73% (99/136)	59% (78/133)	0.0145^b^
**BMI (kg/m^2^),** mean ± SD (*n*)	30.3 ± 5.7 (136)	29.9 ± 5.5 (133)	0.6212^a^
**NYHA (if assessed)**			
ȃClass I	20% (15/76)	26% (21/81)	0.3566^b^
ȃClass II	61% (46/76)	54% (44/81)	0.4321^b^
ȃClass III	20% (15/76)	20% (16/81)	0.9980^b^
**Largest LA diameter (mm)**			
ȃMean ± SD (*n*)	47.7 ± 10.5 (112)	46.5 ± 6.9 (108)	0.3006^a^
**LV ejection fraction (%)**			
ȃMean ± SD (*n*)	56.3 ± 6.9 (128)	56.6 ± 7.3 (129)	0.6570^a^
**Prior and concomitant medications**			
ȃClass 1 AAD	20% (26/132)	17% (21/127)	0.5093^b^
ȃClass 2 AAD	79% (104/132)	77% (98/127)	0.7527^b^
ȃClass 3 AAD	33% (44/132)	28% (36/127)	0.3852^b^
ȃClass 4 AAD	4% (5/132)	4% (5/127)	1.0000^c^
**AF indication at baseline**			
ȃParoxysmal	38% (51/136)	36% (48/133)	0.8106^b^
ȃPersistent	57% (78/136)	59% (78/133)	0.8298^b^
ȃUnspecified	5% (7/136)	5% (7/133)	0.9658^b^

From *t*-test.

From Chi-square test.

From Fisher’s exact test when Cochran’s rule is not met.

Note: All *P*-values displayed are two-tailed and not from pre-specified hypothesis testing and are displayed for information only.

The procedural data are summarized in *Table 2*. There were no significant differences between arms for any of the evaluations performed, except for the ‘Total Rotor Ablation RF Time’. To participate in the study, confirmation of reconnection of one or more PVs was required. There was no significant difference in the number of PV reconnections between the FIRM-guided (2.2 ± 1.0) and Conventional (2.4 ± 1.4) arm (*P*-value = 0.1989). Mean RF time spent on PVI (re-isolation) was 29.5 ± 30.0 min in the FIRM-guided arm and 36.5 ± 32.1 min in the Conventional arm (*P*-value = 0.09). In the FIRM-guided arm, the mean RF time spent ablating rotors was 20.0 ± 20.4 min. The ‘Other Ablation’ category included any RF time spent ablating outside the PVI and Rotor ablation. The mean time spent on other RF ablation was 7.5 ± 27.6 and 6.8 ± 14.3 min in the FIRM and Conventional arms, respectively.

**Table 2:** Procedural data (intent to treat population)

Parameter	FIRM-guided (*N* = 134)	Conventional (*N* = 131)	*P*-value^a^
Number of PVs that had reconnected prior to ablation	2.2 ± 1.0 (109)	2.4 ± 1.4 (102)	0.1989
Total PVI RF time, min, mean ± SD (*n*)	29.5 ± 30.0 (115)	36.5 ± 32.1 (110)	0.0902
Total rotor ablation RF time, min, mean ± SD (*n*)	20.0 ± 20.4 (94)	N/A	N/A
Total other ablation RF time, min, mean ± SD (*n*)	7.5 ± 27.6 (117)	6.8 ± 14.3 (112)	0.8063
Total RF time, min, mean ± SD (*n*)	47.3 ± 29.3 (79)	42.8 ± 33.4 (110)	0.3327

From *t*-test.

Ablation outside of PVI-zones (except Rotor areas) in the FIRM-guided vs. the Conventional arm did not differ significantly: RA-Isthmus Line/CTI 9.0% vs. 15.3%, caval vein isolation 1.5% vs. 0.8%, non-PV trigger 2.2% vs. 3.8%, other [non-PV/non-wide antral circumferential ablation (WACA)] 11.9% vs. 13.0%.

**Figure 2:**
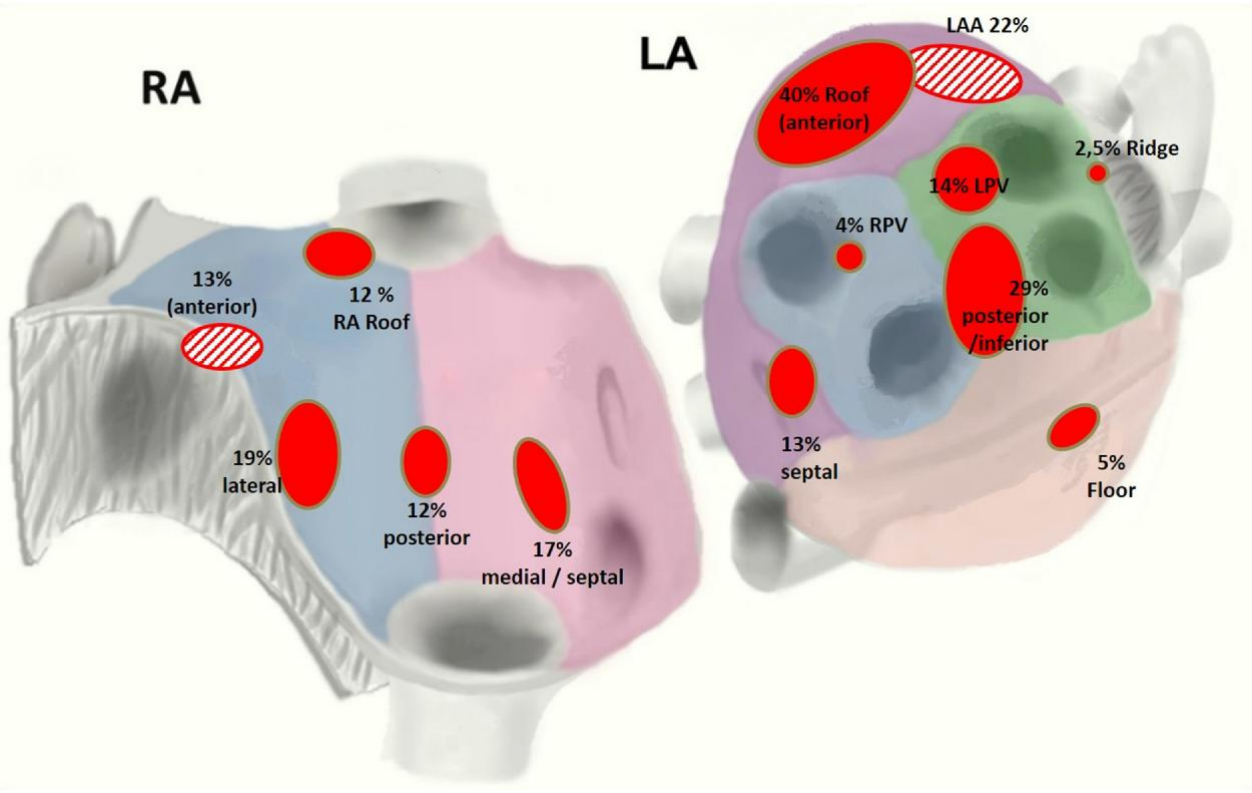
Location of rotors (intent to treat population).

*Figure 2* shows the percentage of subjects (ITT Population) with at least one identified rotor in each region of the right and left atria. In subjects with at least one identified rotor, the total mean number of rotors were 2.4 ± 2.4 (86) in the left atrium and 2.2 ± 1.8 (49) in the right atrium. There were five subjects with no identified rotors in the left atrium and 43 subjects with no identified rotors in the right atrium. When all ITT subjects are considered, the total mean number of rotors were 2.1 ± 2.4 (98) in the left atrium and 1.1 ± 1.7 (98) in the right atrium.

### Primary effectiveness endpoint results

**Figure 3:**
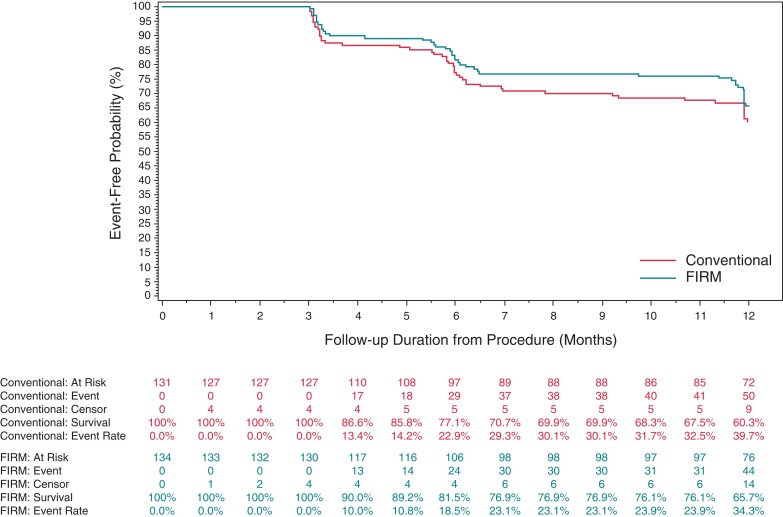
Single-procedure freedom from atrial fibrillation/atrial tachycardia/AFL recurrence at 12 months—overall results.

The primary effectiveness endpoint was defined as single-procedure freedom from AF/AT/AFL recurrence at 3-12 months post procedure. Single procedure freedom from AF/AT/AFL recurrence at 12-months was achieved in 63.3% (76/120) and 59.0% (72/122) of subjects in the FIRM-guided and Conventional arms, respectively (*P*-value = 0.3503). In the subgroup of patients who were indicated to have paroxysmal AF at time of procedure, success was achieved in 76.6% (36/47) and 59.5% (25/42) of subjects in the FIRM-guided and Conventional arms, respectively (*P*-value = 0.1044). In the subgroup of patients who were indicated to have persistent AF at time of procedure, success was achieved in 53.7% (36/67) and 59.5% (44/74) of subjects in the FIRM-guided and Conventional arms, respectively (*P*-value = 0.9341). While there appeared to be a trend towards superiority in the Paroxysmal FIRM-guided arm, there was no significant difference between the two arms in any of these subgroups. The results are summarized in *Table 3*, whereas the Kaplan-Meier-curves are depicted in *Figure 3* and Supplementary material online, appendix figures S2 and S3.

**Table 3:** Primary effectiveness endpoint results (intent to treat population)

Single-procedure freedom from AF/AT/AFL recurrence at 12 months	FIRM-guided (*N* = 134)	Conventional (*N* = 131)	*P*-value (log-rank)
All ITT subjects^a^	63.3% (76/120)	59.0% (72/122)	0.3503
ȃParoxysmal AF subjects	76.6% (36/47)	59.5% (25/42)	0.1044
ȃPersistent AF subjects	53.7% (36/67)	59.5% (44/74)	0.9341
ȃUnspecified AF subjects	66.7% (4/6)	50.0% (3/6)	0.9381

Of the 134 FIRM-guided arm ITT subjects, 14 subjects were reported by the site as having discontinued the study prior to the end of the 12-month observation period without experiencing AF/AT/AFL recurrence. Of the 131 conventional arm ITT subjects, 9 subjects were reported by the site as having discontinued the study prior to the end of the 12-month observation period without experiencing AF/AT/AFL recurrence.

### Primary safety endpoint results

The 10-day safety endpoint was achieved in 93.3% (125/134) and 93.8% (120/128) of subjects in the FIRM-guided and Conventional arms, respectively (*P*-value = 0.8782). The 12-month safety endpoint was achieved in 88.4% (107/121) and 93.4% (113/121) of subjects in the FIRM-guided and Conventional arms, respectively (*P*-value = 0.2194). There were no statistical differences between the study arms in either the paroxysmal or persistent subgroups (*Table 4*).

**Table 4:** Primary safety endpoint results (intent to treat population)

Endpoint description	FIRM-guided	Conventional	*P*-value^a^
**Acute safety: Freedom from procedure-related SAEs through 10 days post-procedure**			
All ITT subjects^b^	93.3% (125/134)	93.8% (120/128)	0.8782
ȃParoxysmal AF subjects	94.0% (47/50)	91.3% (42/46)	0.6118
ȃPersistent AF subjects	92.3% (72/78)	94.7% (71/75)	0.5551
ȃUnspecified AF subjects	100.0% (6/6)	100.0% (7/7)	1.0000
**Long-term Safety: Freedom from procedure-related SAEs through 12-months post-procedure**			
All ITT subjects^c^	88.4% (107/121)	93.4% (113/121)	0.2194
ȃParoxysmal AF subjects	83.0% (39/47)	90.2% (37/41)	0.3085
ȃPersistent AF subjects	91.2% (62/68)	94.6% (70/74)	0.5351
ȃUnspecified AF subjects	100.0% (6/6)	100.0% (7/7)	1.0000

The *P*-value for the acute endpoint used the Chi-Square test. The *P*-value for the long-term endpoint used the Log- rank test.

Of the 134 FIRM-guided arm ITT subjects, 0 subjects were reported by the site as having discontinued the study prior to the end of the 10-day observation period without experiencing AF/AT/AFL recurrence. Of the 131 conventional arm ITT subjects, 3 subjects were reported by the site as having discontinued the study prior to the end of the 10-day observation period without experiencing AF/AT/AFL recurrence.

Of the 134 FIRM-guided arm ITT subjects, 13 subjects were reported by the site as having discontinued the study prior to the end of the 12-month observation period without experiencing AF/AT/AFL recurrence. Of the 131 conventional arm ITT subjects, 10 subjects were reported by the site as having discontinued the study prior to the end of the 12-month observation period without experiencing AF/AT/AFL recurrence.

### Procedural related events

There were 17 procedure-related events, 12 in FIRM-guided arm and 5 in Conventional arm, summarized in *Table 5*. While there were more vascular access site complication events in the FIRM-guided arm, the incidence was not significantly different between the two arms.

**Table 5:** Procedural-related serious adverse events (intent to treat population)

	FIRM-guided events *n*	FIRM-guided subjects (%) (*n*/*N*)	Conventional events *n*	Conventional subjects (%) (*n*/*N*)	*P*-value^a^
Vascular access site complications	6	4.5% (6/134)	1	0,8% (1/131)	0.1202
Pericardial effusion/cardiac tamponade	1	0,7% (1/134)	1	0,8% (1/131)	1.0000
Cerebrovascular accident	1	0,7% (1/134)	1	0,8% (1/131)	1.0000
Esophageal stent insertion	1	0.7% (1/134)	0	0% (0/131)	1.0000
Other	3	2.3% (3/134)	2	1.5% (2/131)	1.0000
**Total**	**12**	**9.0% (12/134)**	**5**	**3.8% (5/131)**	**0**.**0854**

Table 5 shows the procedural-related serious adverse events (SAE's). The main SAE's are listed individually. The bottom row (bold values) shows the sum of all SAE's for each study arm.

All *P*-values by Fisher exact test.

### Secondary endpoint results

Acute effectiveness of the FIRM-guided procedure was evaluated as a secondary endpoint. Success was defined as the ablation and elimination of all arrhythmia sources (rotors) identified by RhythmView. As not all identified rotors were ablated, a secondary analysis was performed to analyze the elimination success of those rotors that were targeted for ablation. Supplementary material online, appendix table S1 summarizes the secondary endpoint results.

### Additional evaluations

Early recurrence of AF/AT was defined as recurrence within the 3-month blanking period. In both arms, the early recurrence rate was low (6.2% in FIRM-guided, 7.1% in Conventional, *P-*value = 0.7507).

## Discussion

### Main findings of the study

RedoFIRM is the first randomized trial assessing the safety and efficacy of FIRM-guided ablation (including Re-PVI), compared with a conventional ablation strategy (mainly Re-PVI) for the treatment of recurrent persistent or paroxysmal AF after an initial AF ablation procedure.

Single procedure freedom from AF/AT/AFL-recurrence at 12-months did not differ significantly and was achieved in 63.3% and 59.0% of subjects in the FIRM-guided and Conventional arms, respectively. While there was a trend towards superiority of the FIRM-guided arm in the paroxysmal subgroup, it was not statistically significant. Ablation outside of PVI-zones (and ablated rotors) in the FIRM vs. the Conventional arm was only performed in a minority of patients and did not differ significantly: RA-Isthmus Line/CTI 9.0% vs. 15.3%, caval vein isolation 1.5% vs. 0.8%, non-PV trigger 2.2% vs. 3.8%, other (non-PV/non WACA) 11.9% vs. 13.0%.

### Driver mechanism of AF

With improved mapping technologies applied to electrophysiological studies of AF in animal models and patients, a repetitive activation pattern (driver mechanism) of AF has been demonstrated.^7^

Using phase analysis, Narayan and colleagues^5^ reported finding putative focal drivers and rotors with spatial stability. However, recent studies from Narayan’s group^8^ and Schotten’s group^9^ demonstrated that phase analysis produces a different mechanistic result than classical activation sequence analysis. The interpretation of phase maps could be limited by artefact due to noise, far-field ventricular signals, and limited resolution with basket catheters.^10–12^

There is an ongoing controversy regarding the impact of rotors and focal sources as a relevant driving mechanism in human persistent AF.

### Focal impulse and rotor modulation guided ablation

The Topera mapping system (now RhythmView, Abbott, Abbott Park, IL) was among the first mapping system to demonstrate rotational drivers during human AF using a computerized algorithm that analyzes the atrial activation acquired with a 64-pole basket catheter and creates 2D focal impulse and ‘rotor’ modulation (FIRM) maps.^13^ The complexity and extent of the mathematical transformation of the raw data left many investigators and clinicians sceptical.

In the CONFIRM trial, freedom from AF was higher in the FIRM-guided arm (82.4%, vs 44.9%; P < .001) after 273 days follow-up. While early data were promising,^14,15^ subsequent data had mixed results.^16,17^ In the recently presented results from the large, randomized REAFFIRM trial (NCT02274857), FIRM + PVI was compared to PVI+ any additional ablation the operator preferred. The trial could not prove any benefit from the FIRM-guided ablation vs the ‘conventional’ strategy with a single procedure 12-month freedom of AF/AT recurrence of 69.3% in the FIRM group and 67.5% in the conventional group.

The recently published FIRMap-AF-study reinforced the importance of PVI in paroxysmal AF patients and demonstrated that FIRM-guided ablation alone (without PVI) is not an effective strategy for treatment of paroxysmal AF in most patients.^18^

### Other technologies to detect drivers for ablation

The recently published UNCOVER AF trial demonstrated that addition of driver ablation to PVI using a noncontact based mapping system produced single procedure success of 72.5%^19^ and the RADAR trial demonstrated that ablation of drivers + PVI also produced a 1-year success of 72%.^20^ Both technologies have to be proven in prospective multicentre randomized trials.

### Conventional strategies in Redo-procedures

In a recent trial the authors described a series of patients undergoing their first repeat ablations for symptomatic, recurrent AF in the contemporary era of contact force-sensing RF catheters.^21^

The main findings were: (i) over 85% of patients at repeat ablation had at least one PV reconnection, with a similar distribution of reconnections in patients with persistent vs. paroxysmal AF; (ii) freedom from atrial arrhythmia at 1-year follow-up after repeat ablation was 66%, similar in those with persistent vs. paroxysmal AF; and (iii) incidence of major complications with repeat ablations was very low.

Non-PV ablation, performed in the majority of patients at repeat ablation, most commonly involved the LA roof, CTI, LA posterior wall, and mitral isthmus. Randomized trials, such as STAR AF II and CHASE-AF, which examined substrate modification, consisting of CFAE ablation and linear ablation lines, did not find further reduction of AF recurrence relative to PVI alone; however, these studies included only persistent AF patients receiving first-time RF ablation. In this study cohort, additional non-PV ablation was not associated with greater 1-year success.

Two randomized controlled trials demonstrated comparable or better efficacies with ablation of low-voltages areas (LVAs) in addition to PVI than with conventional complex-electrogram guided ablation or linear ablation strategies, mainly in persistent AF.^22,23^ However, additional LVA ablation had no beneficial impact on 1-year rhythm outcomes in patients with paroxysmal AF.^1^

## Conclusion

REDO-FIRM did not provide evidence of additional efficacy of FIRM-guided ablation in redo ablation procedures compared to standard ablation. But it is important to mention, that the initial assumptions for the trial design were 40% success in Conventional and 60% in FIRM-guided subjects. That means that the conventional group did much better than expected.

Second, in the FIRM-guided arm, 64.2% and 36.6% of subjects had at least one rotor identified in the left or right atrium, respectively. One might speculate that the full benefit of FIRM-guided procedures may be diluted in this cohort due to 26.9% of FIRM-guided subjects with no rotors identified in either atrium.

In summary the optimal ablation strategy besides Re-PVI in case of reconnected veins in Redo-patients remains unclear. Further research for this patient group is needed and should especially focus on patients, in whom the PV`s are completely isolated at the time of re-ablation.

### Limitations

Implantable loop recorders improve the detection of recurrent AF episodes and AF burden reduction and may represent a better endpoint to measure the success of AF ablation. It is unknown whether differences would have been found with the use of more intensive rhythm monitoring.

Patients were eligible for this study regardless of the type of energy used (RF or cryo) or lesion strategy employed in their first ablation. If the study had been limited to PVI-only ablations in the left atrium, it is possible that more rotors would have been present in all subjects and may have resulted in a difference in outcomes.

Not all subjects, particularly paroxysmal subjects, may have identifiable rotors. However, in subjects where rotors are identified, and successfully ablated, these additional ablations may be beneficial. Since subjects in the Conventional arm were not mapped with the FIRMap diagnostic catheter, it is impossible to know if there would be a difference in outcomes between subjects with rotors identified and ablated vs. those with rotors identified and not ablated.

Enrolment in this study occurred over a period of 3 years, long enough that some operators may have evolved their conventional ablation strategy. However, as subjects were randomized between arms during the entire study, any evolution in strategy is not thought to have impacted one arm more than the other.

## Supplementary material

Supplementary material is available at *Europace* online.

## Supplementary Material

euac122_Supplementary_Data

## Data Availability

The data underlying this article will be shared on reasonable request to the corresponding author.

## References

[1] Masuda M , Asai M, Iida O, Okamoto S, Ishihara T, Nanto K, et al Additional low-voltage-area ablation in patients with paroxysmal atrial fibrillation: results of the randomized controlled VOLCANO trial. J Am Heart Assoc 2020;9:e015927. 32578466 10.1161/JAHA.120.015927 PMC7670527

[2] Haissaguerre M , Jais P, Shah DC, Takahashi A, Hocini M, Quiniou G, et al Spontaneous initiation of atrial fibrillation by ectopic beats originating in the pulmonary veins. N Engl J Med 1998;339:659–666. 9725923 10.1056/NEJM199809033391003

[3] Sanders P , Berenfeld O, Hocini M, Jaïs P, Vaidyanathan R, Hsu L-F, et al Spectral analysis identifies sites of high-frequency activity maintaining atrial fibrillation in humans. Circulation 2005;112:789–797. 16061740 10.1161/CIRCULATIONAHA.104.517011

[4] Hayashi K , An Y, Nagashima M, Hiroshima K, Ohe M, Makihara Y, et al Importance of nonpulmonary vein foci in catheter ablation for paroxysmal atrial fibrillation. Heart Rhythm 2015;12:1918–1924. 25962801 10.1016/j.hrthm.2015.05.003

[5] Narayan SM , Krummen DE, Clopton P, Shivkumar K, Miller JM. Direct or coincidental elimination of stable rotors or focal sources may explain successful atrial fibrillation ablation: on-treatment analysis of the CONFIRM trial (Conventional ablation for AF with or without focal impulse and rotor modulation). J Am Coll Cardiol 2013;62:138–147. 23563126 10.1016/j.jacc.2013.03.021 PMC3703494

[6] Buch E , Share M, Tung R, Benharash P, Sharma P, Koneru J, et al Long-term clinical outcomes of focal impulse and rotor modulation for treatment of atrial fibrillation: a multicenter experience. Heart Rhythm 2016;13:636–641. 26498260 10.1016/j.hrthm.2015.10.031 PMC4762742

[7] Jalife J . Rotors and spiral waves in atrial fibrillation. J Cardiovasc Electrophysiol 2003;14:776–780. 12930260 10.1046/j.1540-8167.2003.03136.x

[8] Zaman JAB , Sauer WH, Alhusseini MI, Baykaner T, Borne RT, Kowalewski CAB, et al Identification and characterization of sites where persistent atrial fibrillation is terminated by localized ablation. Circ Arrhythm Electrophysiol 2018;11:e005258. 29330332 10.1161/CIRCEP.117.005258 PMC5769709

[9] Podziemski P , Zeemering S, Kuklik P, van Hunnik A, Maesen B, Maessen J, et al Rotors detected by phase analysis of filtered, epicardial atrial fibrillation electrograms colocalize with regions of conduction block. Circ Arrhythm Electrophysiol 2018;11:e005858. 30354409 10.1161/CIRCEP.117.005858 PMC6553551

[10] Allessie M , de Groot N. Rebuttal from maurits allessie and natasja de groot. J Physiol 2014;592:3173. 25085971 10.1113/jphysiol.2014.275404 PMC4146365

[11] Pathik B , Kalman JM, Walters T, Kuklik P, Zhao J, Madry A, et al Absence of rotational activity detected using 2-dimensional phase mapping in the corresponding 3-dimensional phase maps in human persistent atrial fibrillation. Heart Rhythm 2018;15:182–192. 28917553 10.1016/j.hrthm.2017.09.010

[12] Berenfeld O , Oral H. The quest for rotors in atrial fibrillation: different nets catch different fishes. Heart Rhythm 2012;9:1440–1441. 22521928 10.1016/j.hrthm.2012.04.029 PMC4292876

[13] Umapathy K , Nair K, Masse S, Krishnan S, Rogers J, Nash MP, et al Phase mapping of cardiac fibrillation. Circ Arrhythm Electrophysiol 2010;3:105–114. 20160178 10.1161/CIRCEP.110.853804

[14] Spitzer SG , Károlyi L, Rämmler C, Scharfe F, Weinmann T, Zieschank M, et al Treatment of recurrent nonparoxysmal atrial fibrillation using focal impulse and rotor mapping (FIRM)-guided rotor ablation: early recurrence and long-term outcomes. J Cardiovasc Electrophysiol 2017;28:31–38. 27766704 10.1111/jce.13110

[15] Miller JM , Kalra V, Das MK, Jain R, Garlie JB, Brewster JA, et al Clinical benefit of ablating localized sources for human atrial fibrillation: the Indiana university FIRM registry. J Am Coll Cardiol 2017;69:1247–1256. 28279291 10.1016/j.jacc.2016.11.079

[16] Mohanty S , Gianni C, Trivedi C, Metz T, Bai R, Al-Ahmad A, et al Impact of rotor ablation in non-paroxysmal AF patients: findings from the per-protocol population of the OASIS trial at long-term follow-up. Am Heart J 2018;205:145–148. 30144981 10.1016/j.ahj.2018.05.021

[17] Gianni C , Mohanty S, Di Biase L, Metz T, Trivedi C, Gökoğlan Y, et al Acute and early outcomes of focal impulse and rotor modulation (FIRM)-guided rotors-only ablation in patients with nonparoxysmal atrial fibrillation. Heart Rhythm 2016;13:830–835. 26706193 10.1016/j.hrthm.2015.12.028

[18] Tilz RR , Lenz C, Sommer P, Roza MS, Sarver AE, Williams CG, et al Focal impulse and rotor modulation ablation vs. pulmonary vein isolation for the treatment of paroxysmal atrial fibrillation: results from the FIRMAP AF study. Europace 2020;23:722–730. 10.1093/europace/euaa378 PMC8139814 33351076

[19] Willems S , Verma A, Betts TR, Murray S, Neuzil P, Ince H, et al Targeting nonpulmonary vein sources in persistent atrial fibrillation identified by noncontact charge density mapping: UNCOVER AF Trial. Circ Arrhythm Electrophysiol 2019;12:e007233. 31242746 10.1161/CIRCEP.119.007233

[20] Reddy V , Choudury S, Sundaram S. Real-time electrogram analysis for drivers of atrial fibrillation (Radar): a multi-center, FDA-IDE, clinical trial of persistent AF. Heart Rhythm Society 40 th Annual late breaking trial session Available at: https://clinicaltrialsgov/ct2/show/NCT03263702 Accessed May. 2019; 10.

[21] Daimee UA , Akhtar T, Boyle TA, Jager L, Arbab-Zadeh A, Marine JE, et al Repeat catheter ablation for recurrent atrial fibrillation: electrophysiologic findings and clinical outcomes. J Cardiovasc Electrophysiol 2021;32:628–638. 33410561 10.1111/jce.14867

[22] Kircher S , Arya A, Altmann D, Rolf S, Bollmann A, Sommer P, et al Individually tailored vs. standardized substrate modification during radiofrequency catheter ablation for atrial fibrillation: a randomized study. Europace 2018;20:1766–1775. 29177475 10.1093/europace/eux310

[23] Yang B , Jiang C, Lin Y, Yang G, Chu H, Cai H, et al STABLE-SR (electrophysiological substrate ablation in the left atrium during sinus rhythm) for the treatment of nonparoxysmal atrial fibrillation: a prospective, multicenter randomized clinical trial. Circ Arrhythm Electrophysiol 2017;10:e005405. 29141843 10.1161/CIRCEP.117.005405

