# Supplementary material for: Randomized evaluation of redo ablation procedures of atrial fibrillation with focal impulse and rotor modulation-guided procedures: the REDO-FIRM study
Source: Europace. 2022 Sep 3;25(1):74–82. doi: 10.1093/europace/euac122 (PMC10103554; doi:10.1093/europace/euac122)
Supplement: euac122_Supplementary_Data [file euac122_supplementary_data.docx]

**SUPPLEMENT TO:**

**Randomized Evaluation of Redo Ablation Procedures of Atrial Fibrillation with Focal Impulse and Rotor Modulation Guided Procedures – The REDO-FIRM study**

Spitzer SG^1^, Miller J^2^, Sommer P^3^, Szili-Torok T^4^, Reddy VY^5^, Nölker G^6^, Williams C^7^, Sarver AE^7^, Wilber DJ^8^

**Online content:**

**Page 2:** Inclusion Criteria

**Page 3 + 4:** Exclusion criteria

**Page 5 – 7:** Adverse Events

**Page 8:** Power and sample size estimation

**Page 9:** Detailed description of the further statistical analyzis

**Page 10:** Appendix Fig. 1: RhythmView’s 6.1 interface

**Page 11:** Appendix Fig. 2 and 3: Single procedure freedom from AF/AT/Afl Recurrence at 12 Months – Results of the Parxysmal (Fig 2) and persistent (Fig3) AF-patients

**Page 12:** Appendix Table 1: Rotor ablation effectiveness

**Page 13:** Additional disclosures

**Page 14:** Investigators

**Inclusion Criteria**

A left atrial diameter < 6.0 cm via transthoracic echo; or <6.5 cm via CT or MRI was required for inclusion. Subjects with clinically significant structural heart disease, pulmonary disease, or history of myocardial infarction in the previous 3 months were excluded from the study. Also excluded were subjects with atrial septal defect closure device, left atrial appendage closure device, prosthetic mitral or tricuspid valve, or permanent pacemaker. Amiodarone must have been discontinued 4-6 weeks prior to ablation and could be re-administered after ablation at the discretion of the Investigator. Class IC antiarrhythmic drugs (AADs) must have been discontinued (5 half-lives) prior to ablation and could be re-administered after ablation at the discretion of the Investigator.

Subjects were required to meet the following inclusion criteria:

1. Male or female 18 – 80 years of age.
2. Has at least one (1) episode of spontaneous persistent or paroxysmal atrial fibrillation documented by rhythm strip/ECG following the most recent ablation.
3. Had one (1) previous AF ablation after January 01, 2013, but NOT within the last 3 months. Detailed documentation of the previous ablation strategy is required.
4. Oral anticoagulation required with either Novel Oral Anticoagulant (NOAC) or Warfarin (in the case of Warfarin, therapeutic INR ≥ 2.0 for at least three weeks prior to randomization) for those subjects who meet two or more of the following criteria:
   1. Age 65 years or older
   2. Diabetes
   3. Coronary artery disease (CAD)
   4. Congestive heart failure
   5. Hypertension with systolic>165 mm Hg
5. Willingness and able to remain on anti‐coagulation therapy for a minimum of 3 months post procedure for all subjects and at least 12 months post procedure if the subject is on anti‐coagulation pre‐procedure or has CHADS2 score ≥ 2 (or CHADs‐Vasc score >1).
6. Left atrial diameter < 6.0 cm via transthoracic echo; or <6.5 cm via CT or MRI up to 6 months pre‐procedure with documented image of largest dimension, or intra‐procedural ICE or atrial angiogram if CT/MRI not available.
7. Willingness, ability and commitment to participate in baseline and follow‐up evaluations without participation in another clinical trial which may confound the results of this study, unless approved by the Sponsor.
8. Signed informed consent.

**Exclusion Criteria**

Subjects must NOT meet any of the following exclusion criteria:

1. Presence of structural heart disease of clinical significance including:
   1. Coronary artery disease with either:
      1. Coronary artery bypass surgery (CABG) within the last six months, or
      2. Stable/unstable angina or ongoing myocardial ischemia
   2. Congenital heart disease where either the underlying abnormality or its correction prohibits or increases the risk of ablation.
2. NYHA Class IV.
3. Ejection fraction < 35% (within previous 6 months).
4. Previous AF ablation within the last 3 months.
5. ASD closure device, LAA closure device, prosthetic mitral or tricuspid valve, or permanent pacemaker.
6. History of myocardial infarction (MI) within the past three (3) months.
7. Contraindication to Heparin and Warfarin/other novel oral anticoagulants (e.g. dabigatran, rivaroxaban, apixaban).
8. Diagnosed atrial myxoma.
9. Any concomitant arrhythmia or therapy that could interfere with the interpretation of the results from this study.
10. Untreatable allergy to contrast media.
11. Severe electrolyte abnormalities at time of the ablation procedure or atrial fibrillation secondary to electrolyte imbalance, thyroid disease, or reversible non‐cardiac cause.
12. Atrial fibrillation from a reversible cause (e.g., surgery, hyperthyroidism, pericarditis).
13. History of pulmonary embolus within one year of enrollment.
14. Acute pulmonary edema.
15. Atrial clot/thrombus on imaging such as on a trans‐esophageal echocardiogram (TEE) performed within 72 hours of the procedure if deemed appropriate by investigator.
16. Any history of a cerebrovascular disease (including stroke or TIA) within the past 6 months.
17. Any anticipation of cardiac transplantation or other cardiac surgery within the next 12 months.
18. Significant pulmonary disease, (e.g. restrictive pulmonary disease, constrictive or chronic obstructive pulmonary disease) or any other disease or malfunction of the lungs or respiratory system that produces severe chronic symptoms and significantly increases risk to sedation or anesthesia.
19. Acute illness or active systemic infection or sepsis.
20. Any history of blood clotting abnormalities or bleeding abnormalities.
21. Life expectancy of less than 12 months.
22. Any Intramural thrombus, tumor, or other abnormality that precludes catheter introduction or safe manipulation.
23. Women known to be pregnant.

**Adverse Events**

Adverse events were classified using the following definitions (EN ISO/FDIS 14155:2010):

Adverse Event (AE) - Any untoward medical occurrence, unintended disease or injury or any untoward clinical signs (including an abnormal laboratory finding) in subjects, whether or not related to the investigational medical device.

Note 1: This includes events related to the procedures involved.

Note 2: For users or other persons this is restricted to event related to the investigational medical device.

Adverse Device Effect (ADE) – Adverse event related to the use of a medical device. This includes:

Any adverse event resulting from insufficiencies or inadequacies in the instructions for use, the deployment, the implantation, the installation, the operation, or any malfunction of the medical device.

Any event that is a result of a use error or intentional misuse.

Serious Adverse Event (SAE) - Adverse Event that:

- Led to death
- Led to a serious deterioration in the health of the subject that either:
  - resulted in a life-threatening illness or injury*, or
  - resulted in a permanent impairment of a body structure or a body function, or
  - required in-patient hospitalization or prolongation of existing hospitalization, or
  - resulted in medical or surgical intervention to prevent life threatening illness or injury or permanent impairment to a body structure or a body function**.
  - led to fetal distress, fetal death or congenital abnormality or birth defect

Note 1:

- *In this context, the term refers to an event in which the subject was at immediate risk of death at the time of the event; it does NOT refer to an event that might have caused death if it were more severe.
- ** For example, if the occurrence of a “catheter insertion site hematoma” or an “AV fistula” requires a blood transfusion and/or surgical repair, it should be considered a serious adverse event.

Note 2:

- Preplanned hospitalizations for pre-existing condition, or a procedure required by the protocol, without a serious deterioration in health, were not considered to be a serious adverse event. For example, repeat ablation for recurrence of AF was not be considered an SAE.
- Emergency room visits that do not result in hospitalization (i.e., an overnight stay) were evaluated for one of the other serious outcomes to determine if they qualified as SAEs.

Serious Adverse Device Event (SADE) - Adverse device effect that has resulted in any of the consequences characteristic of a serious adverse event.

Unanticipated Adverse Device Event (UADE) – Serious adverse device effect which by its nature, incidence, severity or outcome has not been identified in the current version of the risk analysis report.

Unanticipated – AEs not on the list of anticipated adverse events (below) were considered unanticipated.

- Discomfort due to insertion/removal of vascular sheaths beyond what is normally observed
- Ventricular arrhythmia requiring defibrillation
- Hemorrhage and/or hematoma at sheath insertion requiring evacuation or transfusion
- Cardiac tamponade due to perforation
- Extremity weakness, swelling, and/or pain
- Discomfort and/or damage to the skin, muscles, or nerves due to remaining in a supine position for an extended period of time.
- Nerve injury (diaphragmatic paralysis, pyloric spasm, gastric hypomotility)
- Complete AV block
- Air embolism
- Nausea /vomiting
- Allergic reaction
- Headache different from baseline
- Endocarditis
- Hypertension >180 mm Hg systolic (repeated measures)
- Hypotension <80 mm Hg systolic (repeated measures)
- Esophageal-atria fistula
- Hemothorax
- Brief “black out” periods
- Pericarditis
- Shortness of breath/Dyspnea
- Pseudo aneurysm
- Feeling of chest pain, skipped beats, and/or rapid heart rate different from baseline
- Pulmonary vein stenosis
- Damage to skin from prolonged exposure to x-rays
- Radiation injury
- New arrhythmias (not previously documented)
- Renal failure from IV contrast
- Arterial injury requiring intervention
- Respiratory failure
- Thromboembolism
- Stroke/transient ischemic attack
- Local/systemic infection
- Valvular damage
- Pneumothorax
- Pleural effusion
- AV fistula
- Pulmonary edema
- Thrombophlebitis
- Anemia requiring transfusion
- Pulmonary embolism
- Vasovagal reaction
- Myocardial infarction
- New pericardial effusion >1 cm
- Discomfort and/or damage to the skin, muscles, or nerves due to percutaneous access in excess of usual
- Death
- Left heart access via trans-septal puncture has known potential adverse events of: cardiac perforation, cardiac tamponade, and embolic events. Literature reviews have demonstrated that the risk of such events is <1%. [Mullins CE. Transseptal left heart catheterization: experience with a new technique in 520 pediatric and adult patients. Pediatric cardiology. 1983;4:239-45.]

**POWER AND SAMPLE SIZE ESTIMATION**

In the Redo‐FIRM study, the primary effectiveness endpoint is single repeat procedure freedom from AF/AT/AFL in the period 3‐12 months post index ablation procedure. Under discrete, single time point conditions, a simple proportion would be calculated for each group at 12 months and compared, either through traditional categorical analysis techniques (Chi Square tests of independence), or other types of analyses. However, these analyses would not consider the contribution of the period of time prior to the 12‐month endpoint that each subject contributed to their respective group’s “freedom from recurrence of atrial fibrillation at 12 months post index procedure.”

Therefore, the most appropriate means of estimating this proportion at the 12‐month effectiveness endpoint, considering the contribution of subjects failing or censoring (lost to follow‐up, protocol violations, death, etc.) would be a survival analysis approach. Estimates using survival analysis techniques take into account the total contribution for each subject during the period in question. For this analysis, we assume a log‐rank test with constant hazard ratio to test the equality of the survival curves for each group (FIRM‐guided vs. Conventional).

The assumptions used to calculate minimum sample size for the primary effectiveness endpoint of freedom from AF at 3 months post index procedure, are as follows:

**Description Assumptions**

- FIRM Group Freedom from recurrence of AF/AT/AFL 3‐12 months post index ablation: 0.60
- CONVENTIONAL Group Freedom from recurrence of AF/AT/AFL 3‐12 months post index ablation: 0.40
- Probability of Type I error (alpha) (two‐sided test): 0.05
- Power (1‐(Probability of Type II error)): 0.80
- Minimum Sample Size: 134 per group

Since the objective(s) of this study relate to the long‐term safety and effectiveness of FIRM‐guided ablation versus conventional ablation for atrial fibrillation (AF), power and sample size estimates have been calculated relative to those endpoints only. We anticipate enrolling 134 subjects in each treatment arm.

A two‐sided 95.0% continuity corrected confidence interval for the difference between FIRM‐guided proportion, pF, of 0.600 and a Conventional proportion, pC, of 0.400 will extend 0.125 from the observed difference in proportions when the total sample size is 268.

This power and sample size calculation was performed using NQuery Advisor 7.0 in the Microsoft Windows operating system.

**Detailed description of the further statistical analysis**

The secondary acute effectiveness endpoint was defined as elimination of the source of arrhythmias identified in the FIRMap activation map, as indicated by either no evidence of the source immediately post‐procedure or reduction of electrogram amplitude to <0.2mV in region(s) designated by RhythmView.

The 12-month safety endpoint was defined as freedom from procedure‐related serious adverse events (SAEs) (including those related to repeat procedures) within 12 months of the initial procedure. The proportion of successes in each treatment arm was evaluated using both Chi‐Square (Binomial, 1df) and by Kaplan‐Meier survival estimation.

The 10-day primary safety endpoint was defined as freedom from serious adverse events related to the procedure within 10 days of the index procedure. The proportion of success in each treatment arm was evaluated using a Chi‐Square test of independence.

All other analyses were descriptive in nature. Continuous variables were summarized as mean +/- standard deviation and the number of observations. Categorical variables were summarized as the proportion of subjects. Adverse events were summarized as the number of events and the proportion of subjects experiencing the event. P-values were reported descriptively, using t-test for continuous variables and chi-square or Fisher Exact Test for categorical variables depending on whether Cochran’s Rule was met.

**Appendix Figure 1:** RhythmView’s 6.1 interface for analyzing activation maps created using the FIRMap diagnostic catheter, including the stability tool.

From: Spitzer SG et al. Rotor mapping: black box or very simple? The FIRM Approach. Herzschrittmacher Elektrophysiol, 2018. 29(3): p.315-321

**Appendix Figure 2: Single-Procedure Freedom From AF/AT/AFL Recurrence at 12-Months – Results of the Paroxysmal AF-Patients**

**
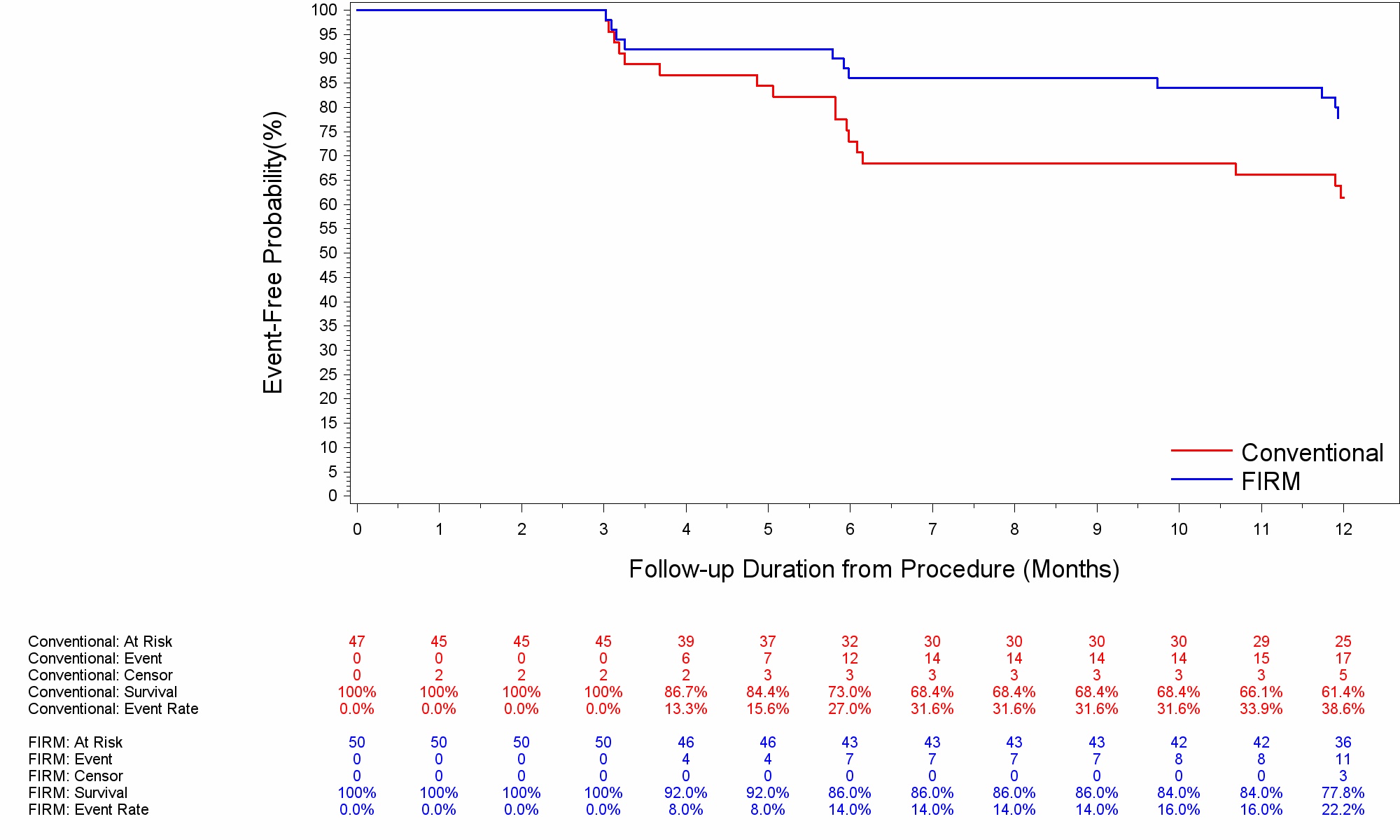
**

**Appendix Figure 3: Single-Procedure Freedom From AF/AT/AFL Recurrence at 12-Months – Results of the persistent AF-Patients**

**
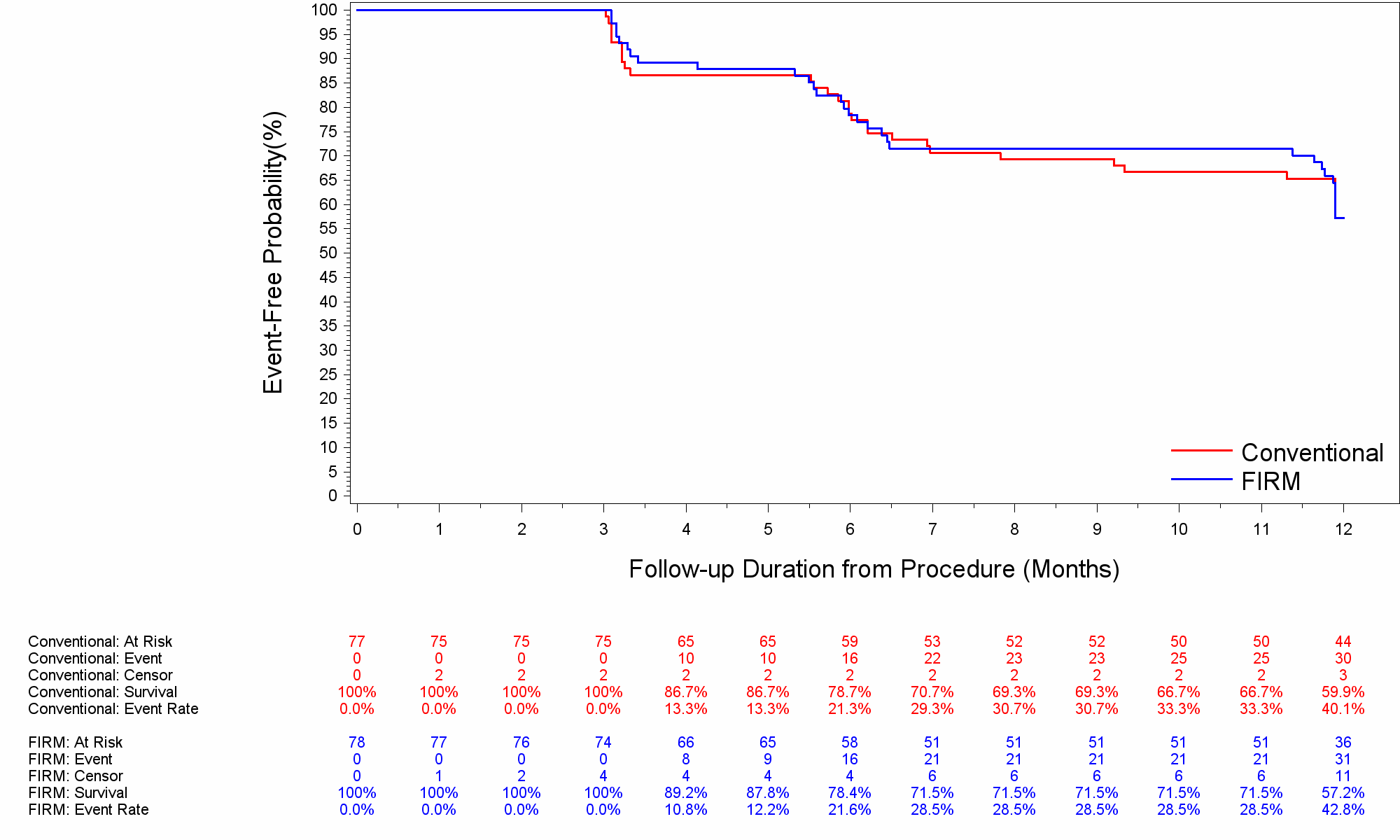
**

**Appendix Table 1: Rotor Ablation Effectiveness**

| **Endpoint Description** | **FIRM-Guided Arm (N=134)** |
| --- | --- |
| **All identified rotors eliminated** | |
| All ITT Subjects | 91.2% (83/91) |
| Paroxysmal AF Subjects | 84.8% (28/33) |
| Persistent AF Subjects | 94.4% (51/54) |
| Unspecified AF Subjects | 100.0% (4/4) |
| **All ablated rotors eliminated** | |
| All ITT Subjects | 92.6% (87/94) |
| Paroxysmal AF Subjects | 87.5% (28/32) |
| Persistent AF Subjects | 94.8% (55/58) |
| Unspecified AF Subjects | 100.0% (4/4) |

**Additional disclosures:**

Unrelated to this manuscript, **VYR** also has these disclosures: Ablacon (Consultant, Equity), Acutus Medical (Consultant, Equity), Affera (Consultant, Equity), Apama Medical (Consultant, Equity), APN Health (Consultant, Equity), Aquaheart (Consultant, Equity), Atacor (Consultant, Equity), Autonomix (Consultant, Equity), Axon Therapies (Consultant, Equity), Backbeat (Consultant, Equity), BioSig (Consultant, Equity), Biosense-Webster (Consultant), BioTel Heart (Consultant), Biotronik (Consultant), Boston Scientific (Consultant), Cardiac Implants (Consultant, Equity), CardiaCare (Consultant, Equity), Cardiofocus (Consultant), Cardionomic (Consultant), CardioNXT / AFTx (Consultant, Equity), Circa Scientific (Consultant, Equity), CoreMap (Consultant), Corvia Medical (Consultant, Equity), Dinova-Hangzhou DiNovA EP Technology (Consultant, Equity), East End Medical (Consultant, Equity), EBR (Consultant), EPD (Consultant, Equity), Epix Therapeutics (Consultant, Equity), EpiEP (Consultant, Equity), Eximo (Consultant, Equity), Farapulse (Consultant, Equity), Fire1 (Consultant), Gore & Associates (Consultant), HRT (Consultant, Equity), Impulse Dynamics (Consultant), Intershunt (Consultant, Equity), Javelin (Consultant, Equity), Kardium (Consultant, Equity), Keystone Heart (Consultant, Equity), LuxMed (Consultant, Equity), Manual Surgical Sciences (Equity), Medlumics (Consultant, Equity), Medtronic (Consultant), Middlepeak (Consultant, Equity), Newpace (Equity), Nuvera (Consultant, Equity), Philips (Consultant), Pulse Biosciences (Consultant), Sirona Medical (Consultant, Equity), Surecor (Equity), Valcare (Consultant, Equity) and Vizaramed (Equity).

**Investigators**

*Europe:*

Dr. Andrea Sarkozy, University Hospital of Antwerp, Belgium

Prof. Dr. Stefan G. Spitzer, Praxisklinik Herz und Gefäße Dresden, Germany

Dr. K.-J. Gutleben (prev. Dr. Georg Nölker), Ruhr University / Bad Oyenhausen, Germany

Prof. Dr. Thorsten Lewalter, Dr. Muller Clinic, München, Germany

Prof. Dr. Philipp Sommer, Leipzig Heart Institute GmbH, Germany

Dr. Jürgen Vogt, Kardiocentrum Frankfurt/Main, Germany

Dr. Tamás Szili-Torok, Medical Center Rotterdam (Erasmus MC), The Netherlands

Dr. Corinna Lenz, Unfallkrankenhaus Berlin, Germany

Dr. Laura Vitali-Serdoz, Fürth Medical Clinic for Heart and Lung Diseases, Germany

Prof. Dr. Roland Tilz, Lübeck University Heart Center, Lübeck, Germany

Dr. Sonia Busch, Klinikum Coburg, Germany

Dr. Gerhard Janssen, Kardiologische Gemeinschaftspraxis am Park Sanssouci, Potsdam, Germany

*USA:*

Dr. Haroon Rashid, Virginia Heart, Falls Church/Arlington, VA

Dr. Vijay Swarup,Arizona Heart Rhythm Center, Phoenix, AZ

Dr. David Wilber, Loyola University Chicago, IL

Dr. Ralph Augostini, Ohio State University, Columbus, OH

Dr. Brad Knight, Northwestern University, Chicago, IL

Dr. Hiroshi Ashikaga, Johns Hopkins Hospital Baltimore, MN

Dr. Grant R. Simons, Englewood Hospital and Medical Center, NJ

Dr. David Callans, University of Pennsylvania, Philadelphia, PA

Dr. Ahmed Osman, Broward Health, Fort Lauderdale ,FL

Dr. Andrew Voigt, University of Pittsburgh Medical Center (UPMC), Pittsburgh, PA

Dr. Anthony Magnano, St. Vincent’s HealthCare, Jacksonville, FL

Dr. Ishu Rao, Ventura Cardiology Consultants, Ventura, CA
